# Supplementary material for: Diclofenac Interacts with Photosynthetic Apparatus: Isolated Spinach Chloroplasts and Thylakoids as a Model System
Source: Plants (Basel). 2024 Aug 8;13(16):2189. doi: 10.3390/plants13162189 (PMC11359304; doi:10.3390/plants13162189)
Supplement: Supplementary file 1 [file plants-13-02189-s001.zip › plants-3138381-supplementary.pdf]

Supplementary Materials to:

Diclofenac Interacts with the Photosynthetic Apparatus: Isolated Spinach Chloroplasts and Thylakoids as a Model System

Monika Majewska [1], Małgorzata Kapusta [2] and Anna Aksmann [1,*]

[1] Department of Plant Experimental Biology and Biotechnology, Faculty of Biology, University of Gdansk, Wita Stwosza 59, 80-308 Gdansk, Poland
[2] Bioimaging Laboratory, Faculty of Biology, University of Gdansk, Wita Stwosza 59, 80-308 Gdansk, Poland

Corresponding Author:
Anna Aksmann
Department of Plant Experimental Biology and Biotechnology, Faculty of Biology, University of Gdansk, Wita Stwosza 59, 80-308 Gdansk, Poland
Email address: anna.aksmann@ug.edu.pl

Figure S1

Kinetics of chlorophyll *a* fluorescence transient in *Spinacia oleracea* chloroplasts treated with diclofenac (DCF) at concentrations of 125, 250, 500, 1000, 2000, or 4000 µM.

Chloroplasts suspension (OD680 = 0.2; 2 mL), prepared as described in "Materials and Methods" section in the main text of the paper, was transfer to the measuring vial. Chlorophyll *a* fluorescence kinetics (OJIP test) was measured using a Handy Pea fluorometer (Hansatech Ltd., Norfolk, UK) run by a Handy Pea software. The following protocol was applied: continuous stirring; saturated light pulse of intensity 3.000 µmol photons × m^{-2} × s^{-1}, measuring time 10 sec. For flurescence parameters calculation the following time marks were applied: T1 = 0.05 msec for O step (F_0); T2 = 0.1 msec; T3 = 0.3 msec for K-step; T4 = 3 msec for J-step; T5 = 30 msec for I step; maximal value of fluorescence signal (the highest point on the fluorescence curve) was considered as P step (F_M).

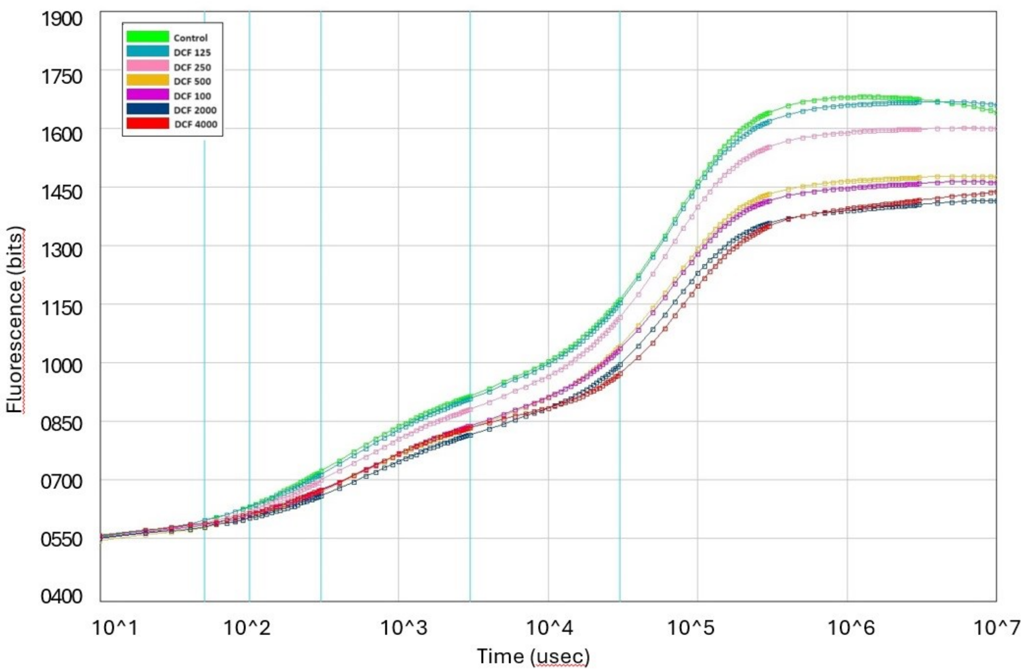

Figure S2

Kinetics of chlorophyll *a* fluorescence transient in *Spinacia oleracea* thylakoids treated with diclofenac (DCF) at concentrations of 1000, 2000, or 4000 µM.

Thylakoids suspension (OD680 = 0.2; 2 mL), prepared as described in "Materials and Methods" section in the main text of the paper, was transfer to the measuring vial. Chlorophyll a fluorescence kinetics (OJIP test) was measured using a Handy Pea fluorometer (Hansatech Ltd., Norfolk, UK) run by a Handy Pea software. The following protocol was applied: continuous stirring; saturated light pulse of intensity 3.000 µmol photons × m^{-2} × s^{-1}, measuring time 10 sec. For flurescence parameters calculation the following time marks were applied: T1 = 0.05 msec for O step (F0); T2 = 0.1 msec; T3 = 0.3 msec for K-step; T4 = 3 msec for J-step; T5 = 30 msec for I step; maximal value of fluorescence signal (the highest point on the fluorescence curve) was considered as P step (FM).

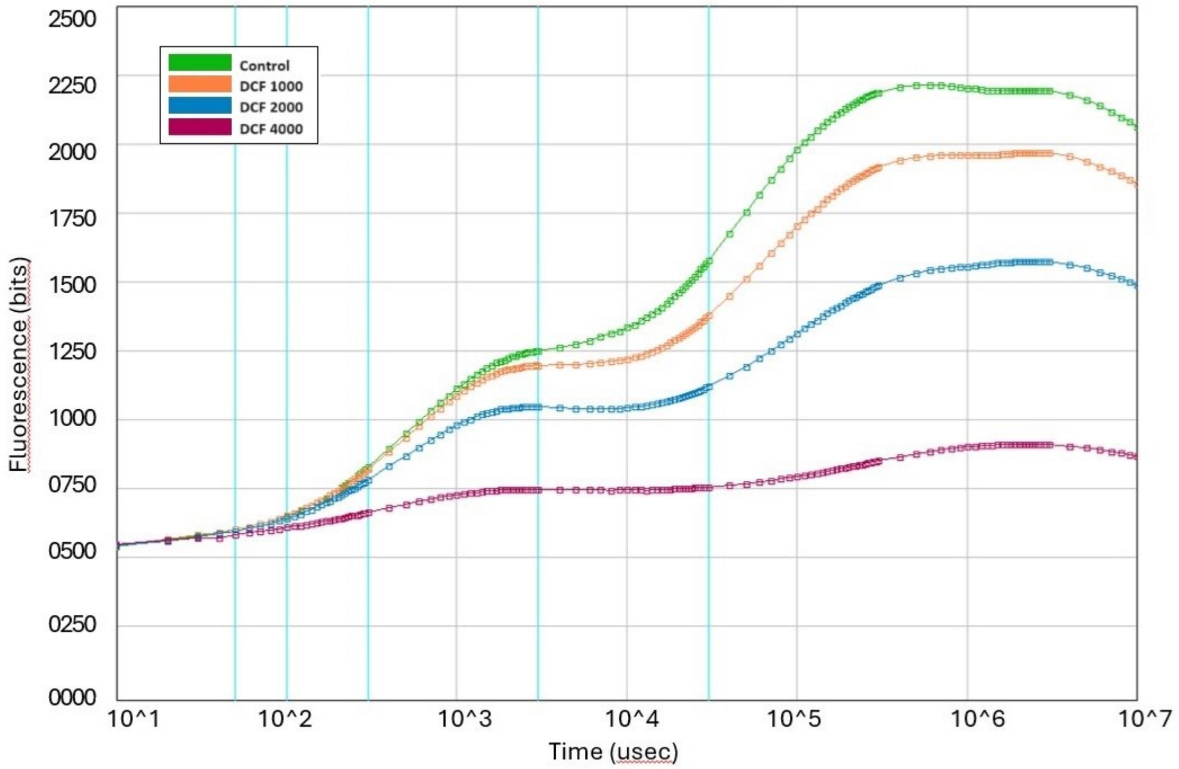

Table S1

The time course of diclofenac (DCF) effects on the chlorophyll *a* fluorescence parameters of *Spinacia oleracea* chloroplasts.

Chloroplasts suspension was prepared as described in "Materials and Methods" section in the main text of the paper. DCF was applied to chloroplasts suspension at concentrations of 125, 1000 or 4000 μM and incubated for 5, 10, 15, 30, 45 or 60 min. Values are means (n = 5) ± SE. Asterisks indicate the significance of differences (Mann-Whitney U-test, $p < 0.05$) between the control (C) and treated chloroplasts.
RC_M—the fraction of active PSII reaction centers; the specific energy fluxes per reaction center for absorption (ABS/RC), trapping (TR_0/RC), electron transport (ET_0/RC), and dissipation (DI_0/RC); φP0 - the maximum yield of primary photochemistry; φD0 - the quantum yield of energy dissipation; ψ0 - the efficiency of the trapped exciton moving the electron further than QA; φE0 - the maximum yield of electron transport; F_V/F_0 - the activity of the water-splitting complex on the donor side of PSII.

		**φP0**	**ψ0**	**φE0**	**φD0**	**ABS/RC**	**TR0/RC**	**ET0/RC**	**DI0/RC**	**Fv/F0**	**RCm**
5 min	C	0,66<br>±0,010<br>**100%**	0,69<br>±0,030<br>**100%**	0,45<br>±0,016<br>**100%**	0,34<br>±0,010<br>**100%**	2,42<br>±0,139<br>**100%**	1,58<br>±0,071<br>**100%**	0,58<br>±0,027<br>**100%**	0,84<br>±0,071<br>**100%**	2,08<br>±0,101<br>**100%**	686,59<br>±48,222<br>**100%**
	DCF125	0,65<br>±0,011<br>**98%**	0,71<br>±0,027<br>**103%**	0,46<br>±0,013<br>**102%**	0,35<br>±0,011<br>**103%**	2,45<br>±0,160<br>**101%**	1,59<br>±0,079<br>**101%**	0,58<br>±0,023<br>**100%**	0,85<br>±0,084<br>**101%**	2,07<br>±0,106<br>**99%**	664,85<br>±42,833<br>**97%**
	DCF1000	0,61<br>±0,019<br>**92%**	0,73<br>±0,015<br>**106%**	0,45<br>±0,015<br>**100%**	0,39<br>±0,019<br>**115%**	2,58<br>±0,127<br>**107%**	1,57<br>±0,058<br>**99%**	0,58<br>±0,014<br>**100%**	1,01<br>±0,093<br>**120%**	1,73<br>±0,162<br>**83%**	575,21<br>±52,572<br>**84%**
	DCF4000	0,48*<br>±0,007<br>**73%**	0,75<br>±0,005<br>**108%**	0,36*<br>±0,003<br>**80%**	0,52*<br>±0,007<br>**153%**	3,46*<br>±0,089<br>**143%**	1,67<br>±0,028<br>**106%**	0,83*<br>±0,017<br>**143%**	1,79*<br>±0,068<br>**213%**	0,98*<br>±0,031<br>**47%**	304,31*<br>±11,268<br>**44%**
10 min	C	0,65<br>±0,014	0,74<br>±0,012	0,48<br>±0,004	0,35<br>±0,014	2,58<br>±0,140	1,67<br>±0,054	0,63<br>±0,019	0,91<br>±0,086	2,03<br>±0,124	624,61<br>±50,125

		**100%**	**100%**	**100%**	**100%**	**100%**	**100%**	**100%**	**100%**	**100%**	**100%**
	DCF125	0,64<br>±0,014<br>**98%**	0,73<br>±0,013<br>**99%**	0,47<br>±0,007<br>**98%**	0,36<br>±0,014<br>**103%**	2,57<br>±0,138<br>**99%**	1,64<br>±0,056<br>**98%**	0,62<br>±0,014<br>**98%**	0,94<br>±0,087<br>**103%**	1,93<br>±0,123<br>**95%**	619,95<br>±47,641<br>**99%**
	DCF1000	0,59<br>±0,023<br>**91%**	0,74<br>±0,012<br>**100%**	0,44<br>±0,017<br>**92%**	0,41<br>±0,023<br>**117%**	2,76<br>±0,110<br>**107%**	1,62<br>±0,053<br>**97%**	0,63<br>±0,006<br>**100%**	1,14<br>±0,100<br>**125%**	1,60<br>±0,181<br>**79%**	498,62<br>±48,428<br>**80%**
	DCF4000	0,44*<br>±0,010<br>**68%**	0,75<br>±0,003<br>**101%**	0,33*<br>±0,006<br>**69%**	0,56*<br>±0,010<br>**160%**	3,83*<br>±0,072<br>**148%**	1,69<br>±0,029<br>**101%**	0,91*<br>±0,020<br>**144%**	2,14*<br>±0,069<br>**235%**	0,84*<br>±0,036<br>**41%**	250,57*<br>±10,244<br>**40%**
15 min	C	0,67<br>±0,021<br>**100%**	0,72<br>±0,011<br>**100%**	0,48<br>±0,010<br>**100%**	0,33<br>±0,021<br>**100%**	2,45<br>±0,149<br>**100%**	1,62<br>±0,043<br>**100%**	0,60<br>±0,028<br>**100%**	0,83<br>±0,109<br>**100%**	2,24<br>±0,198<br>**100%**	713,27<br>±77,283<br>**100%**
	DCF125	0,63<br>±0,015<br>**94%**	0,73<br>±0,013<br>**101%**	0,46<br>±0,008<br>**96%**	0,37<br>±0,015<br>**112%**	2,40<br>±0,253<br>**98%**	1,50<br>±0,14<br>**93%**	0,59<br>±0,056<br>**98%**	0,89<br>±0,123<br>**107%**	1,86<br>±0,127<br>**83%**	585,69<br>±41,910<br>**82%**
	DCF1000	0,58*<br>±0,023<br>**87%**	0,74<br>±0,012<br>**103%**	0,43<br>±0,017<br>**90%**	0,42*<br>±0,023<br>**127%**	2,80<br>±0,093<br>**114%**	1,61<br>±0,044<br>**99%**	0,65<br>±0,006<br>**108%**	1,19<br>±0,097<br>**143%**	1,50*<br>±0,168<br>**67%**	490,24<br>±51,356<br>**69%**
	DCF4000	0,42*<br>±0,014<br>**63%**	0,74<br>±0,004<br>**103%**	0,31*<br>±0,010<br>**65%**	0,58*<br>±0,014<br>**176%**	4,19*<br>±0,139<br>**171%**	1,73<br>±0,022<br>**107%**	0,95*<br>±0,031<br>**158%**	2,46*<br>±0,135<br>**296%**	0,75*<br>±0,043<br>**33%**	228,99*<br>±16,338<br>**32%**
30 min	C	0,64<br>±0,020	0,73<br>±0,012	0,47<br>±0,011	0,36<br>±0,020	2,61<br>±0,134	1,66<br>±0,035	0,66<br>±0,016	0,94<br>±0,106	1,99<br>±0,149	605,71<br>±48,341

		**100%**	**100%**	**100%**	**100%**	**100%**	**100%**	**100%**	**100%**	**100%**	**100%**
	DCF125	0,61<br>±0,019<br>**95%**	0,73<br>±0,017<br>**100%**	0,44<br>±0,011<br>**94%**	0,39<br>±0,019<br>**108%**	2,62<br>±0,126<br>**100%**	1,59<br>±0,038<br>**96%**	0,65<br>±0,006<br>**98%**	1,03<br>±0,101<br>**110%**	1,71<br>±0,131<br>**86%**	547,91<br>±46,016<br>**90%**
	DCF1000	0,55*<br>±0,026<br>**86%**	0,74<br>±0,011<br>**101%**	0,41<br>±0,018<br>**87%**	0,45*<br>±0,026<br>**125%**	3,05<br>±0,136<br>**117%**	1,66<br>±0,032<br>**100%**	0,70<br>±0,017<br>**106%**	1,39<br>±0,134<br>**148%**	1,34*<br>±0,168<br>**67%**	424,78<br>±51,173<br>**70%**
	DCF4000	0,37*<br>±0,008<br>**58%**	0,73<br>±0,005<br>**100%**	0,27*<br>±0,007<br>**57%**	0,63*<br>±0,008<br>**175%**	4,89*<br>±0,090<br>**187%**	1,82*<br>±0,023<br>**110%**	1,03*<br>±0,028<br>**156%**	3,07*<br>±0,091<br>**327%**	0,63*<br>±0,023<br>**32%**	179,52*<br>±6,665<br>**30%**
45 min	C	0,63<br>±0,022<br>**100%**	0,74<br>±0,009<br>**100%**	0,47<br>±0,012<br>**100%**	0,37<br>±0,022<br>**100%**	2,65<br>±0,150<br>**100%**	1,66<br>±0,039<br>**100%**	0,68<br>±0,017<br>**100%**	0,99<br>±0,122<br>**100%**	1,94<br>±0,161<br>**100%**	595,71<br>±53,053<br>**100%**
	DCF125	0,60<br>±0,017<br>**95%**	0,73<br>±0,012<br>**99%**	0,43<br>±0,008<br>**91%**	0,40<br>±0,017<br>**108%**	2,81<br>±0,135<br>**106%**	1,67<br>±0,036<br>**101%**	0,70<br>±0,008<br>**103%**	1,14<br>±0,106<br>**115%**	1,62<br>±0,114<br>**84%**	500,90<br>±41,675<br>**84%**
	DCF1000	0,53*<br>±0,021<br>**84%**	0,74<br>±0,010<br>**100%**	0,39*<br>±0,013<br>**83%**	0,47*<br>±0,021<br>**127%**	3,16<br>±0,147<br>**119%**	1,67<br>±0,039<br>**101%**	0,73<br>±0,011<br>**107%**	1,49<br>±0,133<br>**150%**	1,24*<br>±0,122<br>**64%**	388,10<br>±38,164<br>**65%**
	DCF4000	0,33*<br>±0,016<br>**52%**	0,70<br>±0,018<br>**95%**	0,23*<br>±0,015<br>**49%**	0,67*<br>±0,016<br>**181%**	5,83*<br>±0,136<br>**220%**	1,90<br>±0,076<br>**114%**	1,09*<br>±0,056<br>**160%**	3,93*<br>±0,168<br>**397%**	0,51*<br>±0,031<br>**26%**	141,51*<br>±8,566<br>**24%**
60 min	C	0,61<br>±0,024<br>**100%**	0,73<br>±0,015<br>**100%**	0,44<br>±0,013<br>**100%**	0,39<br>±0,024<br>**100%**	2,83<br>±0,199<br>**100%**	1,71<br>±0,051<br>**100%**	0,71<br>±0,016<br>**100%**	1,12<br>±0,158<br>**100%**	1,74<br>±0,153<br>**100%**	529,24<br>±44,149<br>**100%**

DCF125	0,58 ±0,019 **95%**	0,73 ±0,015 **100%**	0,42 ±0,009 **95%**	0,42 ±0,019 **108%**	2,92 ±0,137 **103%**	1,68 ±0,032 **98%**	0,72 ±0,005 **101%**	1,24 ±0,118 **111%**	1,50 ±0,115 **86%**	466,59 ±34,354 **88%**
DCF1000	0,52 ±0,025 **85%**	0,74 ±0,009 **101%**	0,39 ±0,017 **89%**	0,48 ±0,025 **123%**	3,23 ±0,112 **114%**	1,67 ±0,032 **98%**	0,74 ±0,019 **104%**	1,56 ±0,127 **139%**	1,18 ±0,138 **68%**	364,93 ±40,771 **69%**
DCF4000	0,31* ±0,009 **51%**	0,70 ±0,013 **96%**	0,22* ±0,009 **50%**	0,69* ±0,009 **177%**	6,31* ±0,241 **223%**	1,93* ±0,051 **113%**	1,18* ±0,060 **166%**	4,39* ±0,212 **391%**	0,47* ±0,019 **27%**	126,08* ±6,319 **24%**

Figure S3

The time course of diclofenac (DCF) effects on the chlorophyll *a* fluorescence parameters of *Spinacia oleracea* chloroplasts.

Chloroplasts suspension was prepared as described in "Materials and Methods" section in the main text of the paper. DCF was applied to chloroplasts suspension at concentrations of 125 (A), 1000 (B), or 4000 (C) μM and incubated for 5, 10, 15, 30, 45 or 60 min. Values are presented as % of control. For numerical data and statistics see Table S1.

RC_M—the fraction of active PSII reaction centers; the specific energy fluxes per reaction center for absorption (ABS/RC), trapping (TR_0/RC), electron transport (ET_0/RC), and dissipation (DI_0/RC); φP0 - the maximum yield of primary photochemistry; φD0 - the quantum yield of energy dissipation; ψ0 - the efficiency of the trapped exciton moving the electron further than QA; φE0 - the maximum yield of electron transport; F_V/F_0 - the activity of the water-splitting complex on the donor side of PSII.

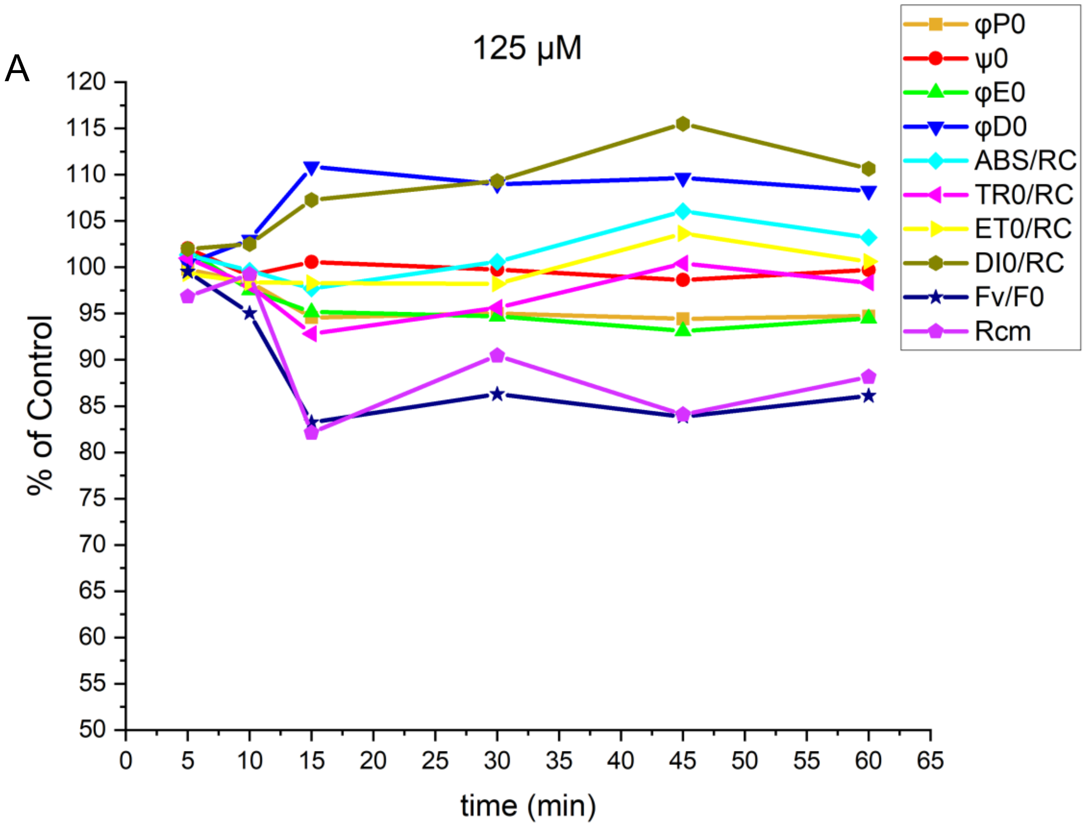

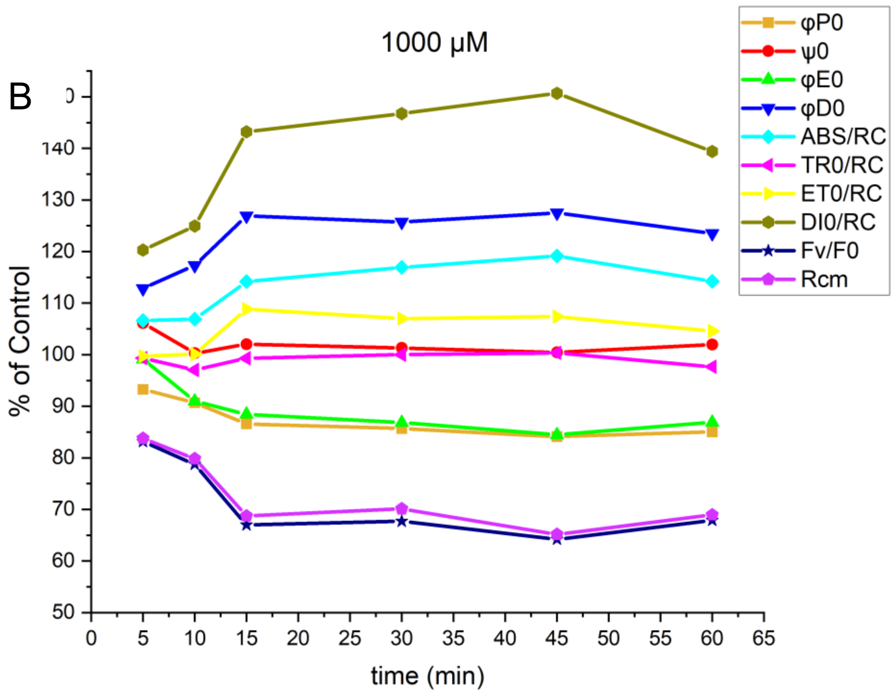
B
1000 µM
% of Control
time (min)
φP0
ψ0
φE0
φD0
ABS/RC
TR0/RC
ET0/RC
DI0/RC
Fv/F0
Rcm

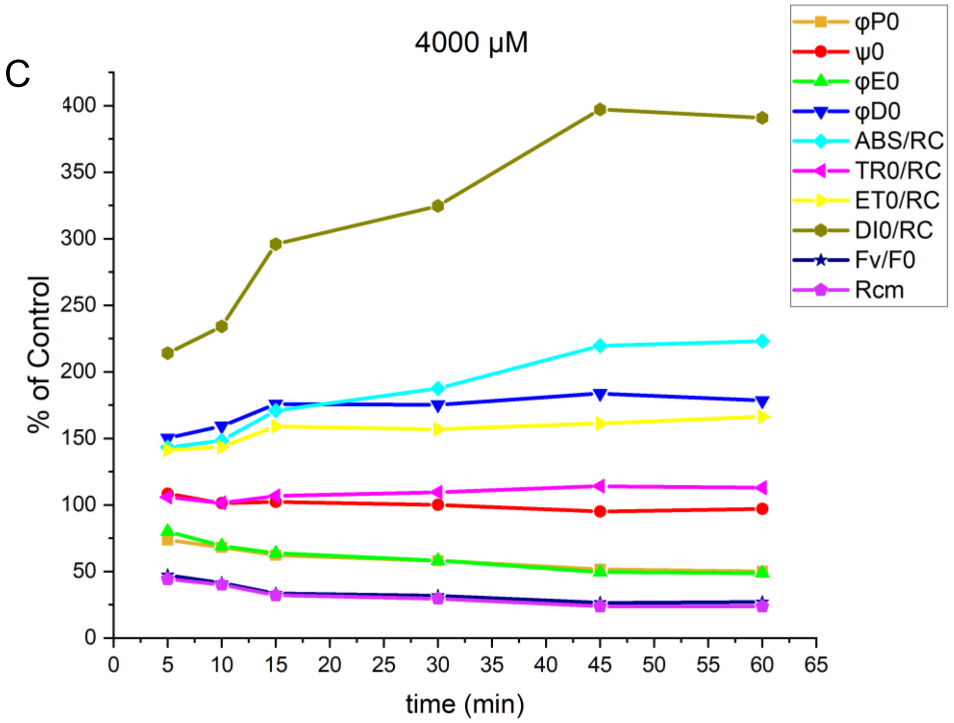
C
4000 µM
% of Control
time (min)
φP0
ψ0
φE0
φD0
ABS/RC
TR0/RC
ET0/RC
DI0/RC
Fv/F0
Rcm
